# MOTA: Network-Based Multi-Omic Data Integration for Biomarker Discovery

**DOI:** 10.3390/metabo10040144

**Published:** 2020-04-08

**Authors:** Ziling Fan, Yuan Zhou, Habtom W. Ressom

**Affiliations:** 1 Department of Biochemistry and Molecular and Cellular Biology, Georgetown University Medical Center, Washington, DC 20057, USA; zf38@georgetown.edu; 2 Department of Oncology, Lombardi Comprehensive Cancer Center, Georgetown University Medical Center, Washington, DC 20057, USA; yz562@georgetown.edu

**Keywords:** multi-omic integration, differential network, metabolomics, transcriptomics

## Abstract

The recent advancement of omic technologies provides researchers with the possibility to search for disease-associated biomarkers at the system level. The integrative analysis of data from a large number of molecules involved at various layers of the biological system offers a great opportunity to rank disease biomarker candidates. In this paper, we propose MOTA, a network-based method that uses data acquired at multiple layers to rank candidate disease biomarkers. The networks constructed by MOTA allow users to investigate the biological significance of the top-ranked biomarker candidates. We evaluated the performance of MOTA in ranking disease-associated molecules from three sets of multi-omic data representing three cohorts of hepatocellular carcinoma (HCC) cases and controls with liver cirrhosis. The results demonstrate that MOTA allows the identification of more top-ranked metabolite biomarker candidates that are shared by two different cohorts compared to traditional statistical methods. Moreover, the mRNA candidates top-ranked by MOTA comprise more cancer driver genes compared to those ranked by traditional differential expression methods.

## 1. Introduction

Statistical and machine learning methods are commonly used in omic studies to find disease biomarker candidates based on differential expression [1,2,3,4,5,6]. However, different biomarker candidates have been reported for different cohorts of the same study, thereby making the candidates less useful as disease biomarkers [7]. One possible way to address this issue is to investigate the association of biomolecules with disease on the basis of the statistical significance not only of changes in their levels but also of changes in their interactions at multiple levels using data from multi-omic studies.

Network-based methods have become an intuitive way to reconstruct biological networks that help investigate the interaction of biomolecules to find disease-associated changes at the system level. For example, relevance networks are a widely used data-driven method to model biological systems due to its simplicity [8]. They measure ‘relevance’ by correlation or mutual information between two biomolecules and set a threshold to determine whether they are relevant or not. However, this method fails to distinguish direct and indirect associations, especially when dealing with high-dimensional omic datasets. This phenomenon is taken into consideration by the Gaussian graphical model (GGM), which estimates the conditional dependency between two features in a dataset by removing the effect brought by others using partial correlation [9,10,11]. Krumsiek et al. used GGM to analyze metabolomic data acquired from a large human population cohort and found that GGM generates rather sparse and robust networks compared to Pearson correlation [12]. They also observed that metabolites from known metabolic reactions are connected by edges with high partial correlation coefficients.

However, interactions between biomolecules within a single layer (e.g., intra-omic interactions among mRNAs) are insufficient to depict a holistic picture of a biological system. Different biological layers are under a tightly coordinated or regulatory relationship. For example, the transcription of mRNA from its DNA template is under the control of its transcription factors which generally are protein molecules. Similarly, metabolic reactions are carried out by enzymes which are also protein molecules or complexes. Therefore, an integrative framework covering interactions over different biological layers help to gain a more comprehensive understanding of biological systems. Huan et al. combined multimodal metabolomic analysis with proteomic and transcriptomic data to identify dysregulated metabolites and pathways [13]. Similar to the idea of constructing intra-omic connections, correlation-based methods can also be applied to investigate inter-omic connections. It has been reported that multivariate methods outperform univariate correlation analyses for calculating the correlation between different datasets [14]. Canonical correlation analysis (CCA) and partial least-squares regression (PLS) are two commonly used multivariate approaches to explore associations of features between two omic studies [15,16]. Whereas CCA aims to find weighted linear combinations of variables maximizing the correlation between two datasets, PLS focuses on covariance rather than correlation.

In this paper, we propose MOTA (Multi-Omic inTegrative Analysis), a network-based integrative method to build a differential coexpression network using multiple omic datasets generated from the same set of samples. The topology of the network and the statistical significance of changes in the levels of features (represented as nodes) are used to rank disease-associated biomolecules. While a coexpression network depicts the pairwise correlations of nodes in the network, the goal of a differential coexpression network, also referred to as a differential network, is to identify the difference in coexpression patterns of nodes in two disparate biological groups (e.g., disease vs. healthy control) [17]. The differential correlation between two biomolecules in disparate biological groups may reflect an altered activating/repressing relationship between the molecules. Recognizing these alterations in the disease-affected group compared to the normal group is a key method in pinpointing dysfunctional regulatory systems and essential disease-associated risk biomolecules. To accomplish this task, we introduce MOTA, which starts by building a differential network based on changes in partial correlation (intra-omic) and canonical correlation (inter-omic) between distinct biological groups. Specifically, we use a regularized generalized version of CCA (rgCCA) that allows us to deal with the large-h small-n problem (h: number of features; n: number of samples) for correlation between features in multiple omic datasets. A MOTA score is calculated considering both the connectivity of nodes (features) in the network and the significance level based on differential expression analysis by statistical methods. We tested MOTA using three sets of multi-omic data obtained by the analysis of sera and liver tissues from three cohorts of hepatocellular carcinoma (HCC) cases and patients with liver cirrhosis (CIRR). The results show that MOTA allows the identification of more overlapping top-ranked metabolite biomarker candidates in two cohorts of the same study compared to *t*-test and iDINGO. Also, mRNA candidates top-ranked by MOTA include more cancer driver genes enriched in cancer-related pathways.

## 2. Methods

### 2.1. Framework of MOTA

**Figure 1:**
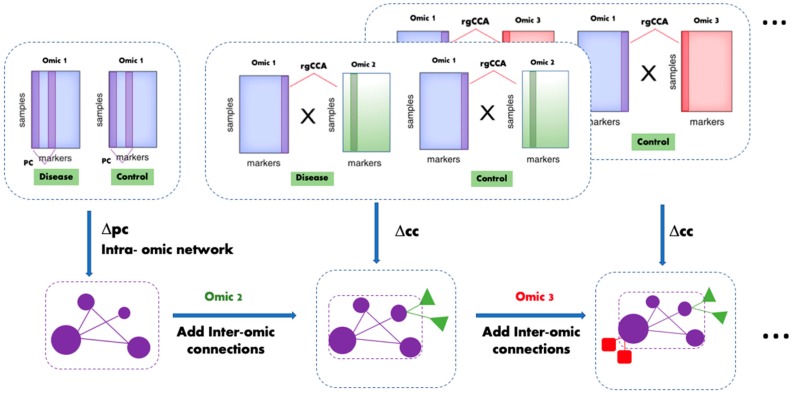
Framework of Multi-Omic inTegrative Analysis (MOTA), demonstrating how an intra-omic network is constructed based on data from Omic 1 and other omic datasets (Omic 2 and Omic 3) to add inter-omic connections to the network. The resulting network allows us to rank disease-associated molecules from Omic 1. Partial correlation (*pc*) is calculated for feature pairs in the Omic 1 dataset, and ∆pc is used to determine intra-omic connections. For other omic datasets, canonical correlation (*cc*) is calculated for feature pairs between Omic 1 and other omic datasets (e.g., Omic 2, Omic 3, etc.), and ∆cc is used to determine inter-omic connections. The MOTA activity score of a node is calculated based on the network topology and statistical significance of the feature represented by the node itself and other features whose nodes (from any of the omic datasets) are connected to it.

Figure 1 depicts the framework of MOTA, which starts by building a differential network using the first omic dataset (denoted as Omic 1 in Figure 1). It calculates the partial correlation (pc) using graphical LASSO for each biomolecule pair in each biological group based on the Omic 1 dataset. Then, it calculates the differential partial correlation (∆pc) to determine intra-omic connections for the network. After building the intra-omic network, MOTA incorporates other available ancillary omic datasets (referred to as Omic 2, Omic 3, etc. in Figure 1) and adds nodes (features) from these datasets. The inter-omic connections are determined by calculating the canonical correlation (cc) for each biomolecule pair based on Omic 1 and another omic dataset (e.g., Omic 2) using rgCCA and computing the differential correlation (∆cc). An activity score is calculated for each node based on its own *p*-value and its connected nodes.

### 2.2. Partial Correlation Calculation Using Graphical LASSO

Graphical LASSO is used to build sparse graphs that mimic the properties of biological networks by adding a LASSO penalty when estimating the inverse covariance matrix (i.e., precision matrix) [18]. The advantage of *pc*, which is calculated using the precision matrix, is that it removes indirect associations caused by other features in the dataset. Graphical LASSO maximizes the following penalized log-likelihood shown in Equation (1):

(1) $$\log(\det(\Theta ))-tr\left(S\Theta \right)-\rho {\left|\left|\Theta \right|\right|}_{1},$$

where Θ is the precision matrix, ***S*** is the sample covariance matrix, *tr* denotes trace, ${\left|\left|\Theta \right|\right|}_{1}$ represents the ℓ1 norm of Θ, which is the sum of the absolute values of all elements in Θ, and ρ is the turning parameter controlling the sparsity of Θ, which is determined by cross validation using the one standard error rule. Precision matrices for both biological groups are calculated using graphical LASSO and partial correlation for each biomolecular pair in each biological group is computed using Equation (2).

(2) $$pc_{ij}=-\frac{\theta_{ij}}{\sqrt{\theta_{ii}\theta_{jj}}},$$

The change in partial correlation for each biomolecular pair between two biological groups (Group 1 and 2) is calculated using Equation (3). A permutation test is used to determine the statistical significance of ∆pc. An edge connecting two nodes is built if ∆pc falls into the 2.5% tails on either end of the empirical distribution curve for $∆\tilde{pc}$.

(3) $$∆pc_{ij}=pc_{ij}^{\left(1\right)}-pc_{ij}^{\left(2\right)},$$

### 2.3. Canonical Correlation Calculation Using Regularized Generalized Canonical Correlation Analysis (rgCCA)

Belonging to multivariate statistical method, rCCA and its generalized formulation, regularized generalized canonical correlation analysis (rgCCA), can be used to associate high-dimensional omic measurements obtained from different platforms (e.g., metabolomics, transcriptomics, proteomics, etc.) [19,20]. In order to determine the correlation method that is best suited for inter-omic connections, we performed a simulation study to compare the performance of Pearson correlation, rCCA, and rgCCA for inferring pre-specified correlations of features in different datasets. The result of the simulation study indicated that rgCCA leads to the lowest error rate for inferring inter-omic connections. Details of the simulation study and results can be found in Supplementary Material (Section S2, Tables S4–S6, Figures S1 and S2). An additional benefit of rgCCA is that it enables the evaluation of associations among biomolecules in more than two omic datasets simultaneously.

Let $X_{1}$, …, $X_{l}$ denote *L* matrices, and $X_{l}$ = {$x_{l1}$, $x_{l2}$,…,$\;x_{lh_{l}}$} denote an *n* ×$\;h_{l}$ matrix (also called a block); *n* is the number of samples; $h_{l}$ is the number of features in the *l*th matrix (dataset). We assume that the columns of $X_{l}s$ are standardized (i.e., a mean of 0 and a variance of 1). In this study, $X_{l}$ represents an omic dataset, and l indexes a specific omic type. rgCCA computes, for each dataset, a weighted composite of variables $y_{l}=X_{l}a_{l}$, l=1,…,L, where $a_{l}$ is a column vector with $h_{l}$ elements, to obtain the optimal solution for the following Equation (4):

(4) $$\underset{a_{1},a_{2},\ldots ,a_{L\;}}{\max}\sum_{l,k=1}^{L}c_{lk}g\left(\mathrm{cov}\left(X_{l}a_{l},X_{k}a_{k}\right)\right)\;s.t.\left(1-\tau_{l}\right)var\left(X_{l}a_{l}\right)+\tau_{l}||a_{l}{||}^{2}=1,\;l=1,\;\ldots ,\;L,$$

where  C is a binary symmetric L×L matrix with each element indicating the network connection between blocks, $c_{lk}=1$ denotes two connected blocks, and $c_{lk}=0$ indicates no connection; g is any continuous convex function used to assign an optimization problem, $\tau_{l}$ is a shrinkage parameter ranging from 0 to 1, $\tau_{l}=1$ yields $||a_{l}{||}^{2}=1,$ which means maximization of the covariance, $\tau_{l}=0$ yields $var\left(X_{l}a_{l}\right)=1,$ which means maximization of the correlation, $0<\tau_{l}<1$ lies between correlation and covariance. In this study, we adopted a ‘horst’ scheme for the g function, which is a scheme leading to the maximization of the sum of the covariances between block components, and $\tau_{l}=0$.

The group-specific canonical correlation ($cc_{ij}^{\left(1\right)}$, $cc_{ij}^{\left(2\right)}$) between two features from two omic datasets (i.e., $x_{ir}$ and $x_{js}$ which are the *i*th feature in Omic *r* and the *j*th feature in Omic *s*) is determined by first projecting $x_{ir}$ and $x_{js}$ onto a low-dimensional space spanned by the first two canonical variants of $X_{r}$ and $X_{s},\;\mathrm{as}\;\mathrm{described}\;\mathrm{in}\;\mathrm{ref}.$ [21]. Then, $cc_{ij}$ is calculated as the inner product between the resulting projected vectors.

Next, the change in *cc* ($∆cc_{ij}$) of the two features (biomolecular pair) between the two biological groups is calculated using Equation (5). We created an edge in the resulting graph if |$∆cc_{ij}$| was above a pre-specified threshold. In order to get a rough estimate of the threshold, we examined different threshold values and determined 0.5 was a reasonable cutoff for datasets considered in this paper. The thresholds and the corresponding assessments we examined are described in Supplementary Material (Section S3). Further investigation is needed to objectively determine the appropriate threshold.

(5) $$∆cc_{ij}=cc_{ij}^{\left(1\right)}-cc_{ij}^{\left(2\right)},$$

### 2.4. MOTA Score Calculation

The network obtained by MOTA consists of intra-omic connections calculated using graph LASSO and inter-omic connections calculated using rgCCA. A MOTA score is calculated for each feature (node) in the intra-omic (seed) network. For example, the *p*-value for node *k* ($p_{k}$) in the seed network is calculated using Student’s *t*-test and then converted to z-score as shown in Equation (6):

(6) $$z_{k}=\emptyset^{-1}\left(1-\frac{p_{k}}{2}\right),$$

where $\phi^{-1}$ is the inverse cumulative distribution function of the standard Gaussian distribution. The MOTA score ($M_{k}$) for node *k* in the seed network is the sum of its own z-score ($z_{k}$) and the summation of all combined z-scores from each omic block connected to it via either intra-omic or inter-omic edges, as shown in Equation (7):

(7) $$M_{k}=z_{k}+\sum_{l=1}^{L}z_{lk},\;k\;\in \;\mathrm{centric}\;\mathrm{dataset}$$

where $z_{lk}$ is calculated using the Stouff’s z-score method through Equation (8), which accounts for the z-scores of all nodes from Omic *l* that are connected to node *k*:

(8) $$z_{lk}\sim \frac{\sum_{i=1}^{m}z_{i}}{\sqrt{m}},$$

where *m* denotes the number of nodes connected to node *k* in Omic *l*, and the $z_{i}$s are their corresponding z-scores.

### 2.5. Multi-Omic Datasets

We tested MOTA using three sets of omic data from three study cohorts. Briefly, blood samples from 87 patients (39 HCC cases and 48 cirrhotic controls) recruited at Tanta University (TU) Hospital and 84 patients (40 HCC cases and 44 cirrhotic controls) recruited at Georgetown University (GU) Hospital [22] were analyzed by targeted metabolomics, glycomics, and proteomics. We refer to these as TU and GU1 datasets, respectively, in Table 1. Additionally, liver tissues from 61 patients (37 HCC cases and 24 cirrhotic controls) recruited at GU Hospital were analyzed by mRNA-seq, miRNA-seq, and metabolomics. These are referred to as the GU2 datasets in Table 1. While all features extracted from the GU1 and TU datasets were used by MOTA, only a subset of the features (549 mRNAs, 125 mRNAs, and 786 metabolites) from the GU2 datasets were selected based on statistical significance (*p*-value < 0.05) between HCC and cirrhotic tissues.

The characteristics of the participants in the three study cohorts are provided in Supplementary Material (Section S1, Tables S1–S3). In this paper, we focused on assessing the ability of MOTA to rank disease-associated metabolites and mRNAs. Using the GU1 and TU datasets, we constructed differential networks and ranked the metabolites based on the MOTA scores of their corresponding nodes. The top-10-ranked metabolites were used to evaluate the performance of MOTA in comparison with those ranked by *t*-test and iDINGO in terms of their reproducibility across the two study cohorts. iDINGO is a tool which adopts the idea of differential network and can be used to integrate multi-omic datasets in an ordered manner [23]. In reference [23], it is emphasized that hubs (nodes with high node degree) are important features of a network topology and may play important roles in disease progression. Therefore, we used the node degree of each node calculated by iDINGO to rank the metabolites. Furthermore, we investigated the top-10-ranked metabolites achieved by concatenating the GU1 and TU datasets. Using the GU2 datasets, we constructed a differential network and ranked mRNAs based on the MOTA scores calculated for their corresponding nodes. The top-30 mRNAs ranked by *t*-test, iDINGO, and MOTA were evaluated on the basis of Gene Ontology (GO) enrichment analysis and the number of top-ranked cancer driver genes.

While the goal of MOTA is to rank disease-associated biomolecules, we assessed the ability of the top-ranked candidates in disease classification using the area under the receiver operating characteristic (ROC) curve (AUC) obtained in distinguishing HCC cases from cirrhotic controls. The results of this assessment can be found in Supplementary Material (Section S4). Briefly, the results showed that, while the features top-ranked by MOTA from one cohort achieved more consistent performance on a different cohort of the same study compared to Student’s *t*-test, their disease classification ability requires a further selection step.

## 3. Results

### 3.1. Ranking Disease-Associated Metabolites

**Figure 2:**
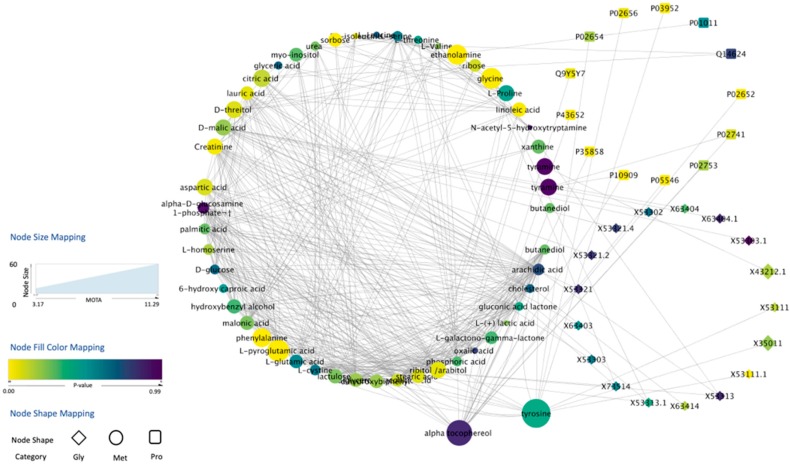
Network constructed by MOTA using the GU1 dataset, which consists of metabolomic, proteomic, and glycomic data, to rank metabolites. The size of a metabolite node is proportional to the corresponding MOTA activity score.

We used MOTA to rank HCC-associated metabolites. Specifically, we calculated MOTA scores for each metabolite in the GU1 metabolomic dataset by integrating it with proteomic and glycomic datasets acquired by analyzing the same set of samples. Also, we applied Student’s *t*-test on GU1 metabolomic dataset to select metabolites with significant changes in their levels between HCC cases and cirrhotic controls. Figure 2 shows the integrated network generated by MOTA, where node color represents the *p*-value (yellower indicates lower *p*-values), and node size indicates the MOTA score (larger size indicates higher MOTA score). Table 2 shows metabolites ranked by *t*-test and MOTA.

In MOTA, the connectivity of node pairs calculated using the idea of differential coexpression renders the edges of the network biologically significant in terms of altered regulatory relationship in distinct biological groups. Once a differential network was constructed, we calculated a MOTA score for each node, assuming that strong candidates tend to be differentially expressed and be surrounded by differentially expressed neighbors. Comparing the ranking results that we obtained by MOTA and *p*-value, two nodes representing tyrosine and α-tocopherol (α-TOH) drew our attention. These metabolites have relatively high *p*-values by Student’s t-test. As illustrated in Figure 2, the primary reason for ranking tyrosine high is that tyrosine has a number of inter-omic connections with low-*p*-value proteins and glycans. Previous work has shown the association of O-glycosylation on tyrosine residues (it can also happen in other amino acids) of proteins with liver cancer [24]. Similarly, connections with multiple proteins are the main reason for increasing α-TOH’s ranking. Through literature review, we found associations of α-TOH with its connected proteins in Figure 2. For example, a previous work has reported a direct physical interaction between α-TOH and Apolipoprotein A-II (P02652) involved in liver lipid metabolism [25]. As shown in Figure 2, α-TOH is also connected with C-reactive protein (CRP, P02741), which is a marker of inflammation. Several previous studies have shown the anti-inflammatory effect of α-TOH [26,27] and indicated a biological relationship between these two proteins. These examples show that MOTA has a promising capability to pinpoint disrupted regulatory relationships between biomolecules by a pathophysiological condition and highlight important risk molecules. Furthermore, researchers could take advantage of MOTA as a hypothesis-generating tool by further investigating the edges or clusters created by MOTA, thereby creating the opportunity to better understand disease mechanisms.

While biomolecules with statistically significant changes in their levels could be associated with a disease, they might be merely the ultimate phenotype which can be detected rather than the disease origins. Reproducibility is highly desired to uncover the molecular mechanism of disease and to develop biomarkers applicable to a wide population in spite of inevitable inherent cohort disparities [28]. Table 3 presents a comparison of feature ranking results obtained by analysis of the TU, GU1, and combined TU and GU1 datasets using *t*-test, iDINGO, and MOTA. To combine the TU and GU1 datasets, we retained features (35 metabolites, 100 proteins, and 82 glycans) that were present in both datasets. We analyzed these three datasets using iDINGO in the order of glycomics, proteomics, and metabolomics to rank metabolites based on their node degree. We ran iDINGO with this order based on the description of the method considering a biological regulatory logic that glycans affect protein functions which have influence on metabolic reactions. As illustrated in Table 3, while only two common metabolites were in the top 10 ranked by *t*-test and iDINGO across the TU, GU1, and combined TU and GU1 datasets, four common metabolites were included in the top 10 ranked by MOTA. The number of overlapped metabolites was consistent between individual cohorts and the combined cohort. Examining the TU network created by MOTA, we can see that the reasons for increased ranking of tyrosine and α-TOH are the same as those previously mentioned for GU (tyrosine’s connection with low *p*-value glycan molecules and α-TOH connection with low *p*-value protein molecules). This finding reveals that differential coexpression seems to be a stable pattern in different cohorts of the same study and can be mined by MOTA. By considering both topology of a differential network and differential expression, MOTA contributes to ranking highly biologically significant biomarker candidates that are reproducible across different cohorts of the same study, whereas iDINGO or Student’s *t*-test consider only one of the two aspects.

### 3.2. Ranking Disease-Associated Genes

Cancer is a genetic disease where changes to genes cause malfunctions in the affected cells and further lead to various phenotypes of the disease [29]. Genetic changes, also referred to as mutations, can be classified into multiple categories (silent mutations, missense mutations, nonsense mutations, and frameshift mutations) and affect downstream biological functions by different mechanisms. Gene transcription is affected by mutations in DNA sequences and acts as a medium propagating the effects of genetic mutations further downstream biological processes. Therefore, transcriptomic studies followed by differential expression analysis have become an important strategy for biomarker discovery or to locate essential disease risk genes for further mechanistic studies. Also, researchers can take advantage of pathway analysis or GO analysis using selected differentially expressed genes to obtain a higher level (pathway level or gene function level) of understanding to the disease. However, it has been reported that sometimes the pathway enrichment analysis performed using genes selected by this method only yields non-specific biologic processes [30]. To this end, we investigated the genes selected by MOTA and assessed their biological significance compared to other traditional differential expression analyses.

We used MOTA to rank mRNAs based on the GU2 datasets by integrating the mRNA profiling data with metabolomic and miRNA profiling data generated from the same cohort of samples. We used DESeq 2 [31] to calculate the *p*-values for each mRNA and to rank the mRNAs. Furthermore, we analyzed the three omic datasets using iDINGO in the order of miRNA, mRNA, and metabolite to rank mRNAs. The GO analysis [32] was done using the top-30 genes prioritized by the three methods. As shown in Table 4, none of the ontology terms obtained based on mRNAs selected by DESeq2 is statistically significant; the top-ranked terms by raw *p*-value seem to have a tangential relationship with cancer. On the other hand, the top-30 genes ranked by iDINGO and MOTA are enriched in 7 and 10 gene functions, respectively. From the GO analysis, we can see that iDINGO ranked in the top-30 mRNAs related to basic cell functions, such as nucleosome or chromatin assembly and DNA packaging. On the other hand, MOTA ranked in the top 30 mRNAs that are related to more specific pathways or cell functions, such as regulation of protein kinase B (PKB) signaling and elastic fiber assembly, which are well studied biological processes involved in cancer development or even liver cancer specifically [33,34].

The GO analysis results showed the capability of MOTA to rank important genes which might directly contribute to cancer development rather than merely vary in expression as a result of the disease. Genetically, mutations of a gene, which are the origin and cause of cancers, do not necessarily lead to a significant expression change of the mRNA transcribed from its DNA template. For example, some ‘missense mutations’ play a role in carcinogenesis by changing protein functions rather than resulting in a quantitative change in gene expression. This maybe the main reason why genes selected by significance analysis are not enriched in important cancer-related processes. On the other hand, MOTA ranks high important genes by connecting them with the biomolecules (miRNAs, proteins, metabolites, etc.) affected by the genetic changes on the basis of differential coexpression. To this end, an interesting question is whether MOTA is able to identify more cancer driver genes. We compared genes in lists obtained by different ranking methods with genes curated in the Sanger Cancer Gene Census [35]. Each gene in the lists possesses a documented activity relevant to cancer, along with evidence of mutations in cancer which change the activity of the gene product in a way that promotes oncogenic transformation. Table 5 shows that the list of genes top-ranked by MOTA contains more cancer driver genes compared to lists obtained by other methods. From these results, we conclude that MOTA can help identify essential disease risk genes involved in important processes of cancer development.

## 4. Discussion

We compared the performance of MOTA to that of iDINGO in terms of number of overlaps between top-ranked metabolites in the GU1 and TU datasets. We found that MOTA identified more overlapping metabolites compared to Student’s *t*-test and iDINGO. Note that the goal of iDINGO is to locate edges representing altered correlation relationships between feature pairs in a differential network rather than ranking candidate biomarkers. We used node degree of each node calculated by iDINGO to rank metabolites in the GU1, TU, and combined GU and TU datasets and compared the results obtained by MOTA to those obtained using the other methods. In terms of activity score calculation, MOTA considers both statistical significance and topology of a differential network. Our results indicate that considering both of these two aspects helps overcome inherent cohort disparities and provides more reproducible results when examining different cohorts of samples.

We performed a GO analysis to further understand the meaning of genes selected by different methods. As shown in Table 4, the GO terms generated using genes top-ranked by iDINGO, which emphasizes hub genes from a differential network, are related to nucleosome and chromatin assembly or DNA packaging, which are closely related to DNA replication. Under the scenario of cancer development, specifically HCC in this study, this result indicates that many genes involved in the process of DNA replication, which is a primary phenotypic change in cancer development, may play an important role in HCC development. On the other hand, MOTA went one step further compared to iDINGO by considering the statistical significance of nodes (differential expression) along with the differential correlation between nodes. Except for terms related to extracellular matrix (ECM) and PKB signaling, which are well recognized in multiple cancer types, another GO term, “elastic fiber assembly”, drew our attention. This is an interesting finding, since the biological process related to it is liver-specific, and previous studies have reported the relationship between liver fibrosis/HCC and elevated elastic fiber biosynthesis [33].

One of the shortcomings of MOTA is its relatively weak performance in disease classification. We speculate that a potential reason is that MOTA is focused on ranking features instead of selecting a parsimonious set of features that leads to improved classification accuracy or features with the most significant difference between disease and control groups. Further investigation is needed to have a comprehensive ranking method that considers the classification performance or to select a set of features from the top-ranked ones with the highest classification accuracy. Also, MOTA accomplishes multi-omic integration by starting with a network from the first omic dataset (Omic 1 in Figure 1) and mapping features (nodes) from other omic datasets (e.g., Omic 2 and Omic 3) onto the network. Future work will focus on investigating not only the interactions among features in Omic 1 vs. Omic 2, and Omic 1 vs. Omic 3 as in Figure 1, but also the interactions among features in Omic 2 vs. Omic 3. For instance, we ranked metabolites based on metabolite–metabolite, metabolite–glycan, and metabolite–protein associations. However, glycosylation, which is reflected by associations between protein molecules and glycans, also plays an important role in biological regulation. Further improvement of MOTA will take this into consideration.

In terms of computing time, MOTA took around 2 min on a Mac computer with Intel Core i5 CPU, 8G RAM, and 256G SSD hard drive to analyze the GU1 and TU datasets that include 53 and 66 metabolites, respectively, and around 200 features from other omic datasets. It took 51 min to analyze the GU2 dataset, which involves 549 mRNAs and 900 features from other omic datasets. Note that MOTA’s running time is mainly determined by the size of the omic dataset whose biomolecules are to be ranked, because of the permutation test (1000 times during testing) that requires a considerable amount of running time.

## 5. Conclusions

In this paper, we introduce MOTA, a network-based method for ranking candidate disease biomarkers. Using three sets of multi-omic data representing three different cohorts, we demonstrated that MOTA allows the identification of more top-ranked biomarker candidates that are shared by two cohorts compared to *t*-test and iDINGO. Also, the networks constructed by MOTA allow the evaluation of the biological significance of biomarker candidates. Moreover, MOTA ranks higher cancer driver genes compared to other traditional differential expression methods. GO analysis result showed that genes ranked high by MOTA were enriched for gene functions that are closely related to carcinogenesis.

## Figures and Tables

**Table 1:** Multi-omic datasets acquired from three cohorts. The number of features in each omic dataset and the number of serum and tissue samples analyzed by multi-omic approaches are shown. HCC, hepatocellular carcinoma, CIRR, cirrhosis, TU, Tanta University, GU, Georgetown University.

Datasets	Omic Studies (No. of Features)	Serum		Tissue	
		HCC	CIRR	HCC	CIRR
TU Datasets	Metabolomics (66) Glycomics (82) Proteomics (100)	39	48		
GU1 Datasets	Metabolomics (53) Glycomics (82) Proteomics (101)	40	44		
GU2 Datasets	Metabolomics (3672) mRNA profiling (27,523) miRNA profiling (2543)			37	24

**Table 2:** Ranking of metabolites in TU datasets. The *p*-value is calculated using Student’s *t*-test.

Feature	*p*-Value	Rank	MOTA	Rank
tyrosine	0.42	36	11.29	1
alpha tocopherol	0.85	50	10.24	2
pyroglutamic acid	0.01	4	8.96	3
glycine	0.01	5	8.62	4
ethanolamine	0.00	1	8.34	5
phenylalanine	0.01	2	7.92	6
citric acid	0.13	16	7.42	7
threitol	0.08	12	7.27	8
tyramine	0.95	53	7.23	9
aspartic acid	0.08	13	7.18	10
ribitol /arabitol	0.06	10	7.08	11
creatinine	0.02	7	7.01	12
malic acid	0.22	20	7.00	13
Proline	0.45	38	7.00	14
lactulose	0.26	23	6.43	15
linoleic acid	0.02	6	6.42	16
hydroxybenzyl alcohol	0.34	33	6.40	17
malonic acid	0.26	24	6.34	18
xanthine	0.29	29	6.30	19
sorbose	0.01	3	6.26	20
myo-inositol	0.31	30	6.23	21
stearic acid	0.08	11	6.20	22
diglycerol	0.21	19	6.18	23
lauric acid	0.06	8	6.18	24

**Table 3:** Biomarker candidates overlapping between the GU1, TU, and combined TU and GU1 datasets ranked by *t*-test, iDINGO, and MOTA.

Rank	GU1 Cohort	TU Cohort	GU1+TU Cohort	No. of Overlaps
Ranking using **Student *t*-Test (*p*-Value)**				
1	**ethanolamine**	glutamic acid	ethanolamine	2
2	phenylalanine	lactic acid	**sorbose**	
3	**sorbose**	alpha tocopherol	citric Acid	
4	pyroglutamic acid	valine	isoleucine	
5	glycine	**ethanolamine**	threitol	
6	linoleic acid	alpha-D-glucosamine 1-phosphate	ribose	
7	creatinine	norvaline	malic acid	
8	lauric acid	citric Acid	phenylalanine	
9	ribitol /arabitol	norleucine	stearic acid	
10	threitol	**sorbose**	trans-aconitic acid	
Ranking using **iDINGO**				
1	linoleic acid	norvaline	valine	2
2	**isoleucine**	cystine	ethanolamine	
3	leucine	**sorbose**	butanediol	
4	proline	tagatose	ribose	
5	ethanolamine	**isoleucine**	glycine	
6	valine	trans-3-hydroxy-L-proline	**sorbose**	
7	glutamic acid	N,N-dimethyl-1-4-phenylenediamine	tyrosine	
8	**sorbose**	cholesterol	malic acid	
9	aspartic acid	butanediol	**isoleucine**	
10	glycine	arachidic acid	tagatose	
Ranking using **MOTA**				
1	**tyrosine**	**alpha tocopherol**	**alpha tocopherol**	4
2	**alpha tocopherol **	**tyrosine**	**ethanolamine**	
3	pyroglutamic acid	**ethanolamine**	glycine	
4	glycine	creatinine	lactic acid	
5	**ethanolamine **	**tyramine**	creatinine	
6	phenylalanine	mimosine	**tyrosine**	
7	citric acid	lactic acid	cholesterol	
8	threitol	cholesterol	**tyramine**	
9	**tyramine **	threitol	citric Acid	
10	aspartic acid	ribose	isoleucine	

Note: Metabolite candidates that appeared in the top-10 ranked lists of all three cohorts are highlighted with the same color.

**Table 4:** Top-5 significant Gene Ontology (GO) terms based on genes selected by DESeq2, iDINGO, and MOTA.

	DESeq2		iDINGO		MOTA	
No. of GO Terms with FDR < 0.05	0		7		10	
	GO Terms	FDR (*p*-Value)	GO Terms	FDR (*p*-Value)	GO Terms	FDR (*p*-Value)
Gene	chromatin organization	1.0 (1.03 × 10^−4^)	chromatin assembly	0.014 (1.73 × 10^−6^)	extracellular matrix organization	1.27 × 10^−5^ (8.0 × 10^−10^)
	kidney development	1.0 (2.31 × 10^−4^)	nucleosome assembly	0.014 (8.69 × 10^−6^)	extracellular structure organization	1.90 × 10^−5^ (2.4 × 10^−9^)
	renal system development	1.0 (2.88 × 10^−4^)	nucleosome organization	0.016 (3.93 × 10^−6^)	positive regulation of protein kinase B signaling	7.56 × 10^−4^ (1.43 × 10^−7^)
	nucleosome assembly	1.0 (3.28 × 10^−4^)	Chromatin assembly or disassembly	0.019 (3.48 × 10^−6^)	cell chemotaxis	1.48 × 10^−3^ (6.56 × 10^−7^)
	urogenital system development	1.0 (4.46 × 10^−4^)	DNA packaging	0.214 (6.73 × 10^−6^)	elastic fiber assembly	1.58 × 10^−3^ (5.98 × 10^−7^)

**Table 5:** Number of cancer driver genes selected using DESeq2, iDINGO, and MOTA.

Top *k*	DESeq2	iDINGO	MOTA
Top 10	1 (*ID3*)	0	2 (*FGFR2*, *PDGFRA*)
Top 50	3 (*ID3*, *PDGFRA*, *HIST1H3B*)	1 (*PDGFRA*)	6 (*FGFR2*, *PDGFRA*, *ID3*, *CDH1*, *SMO*, *EPAS1*)
Top 100	4 (*ID3*, *PDGFRA*, *HIST1H3B*, *HSP90AB1*)	3 (*PDGFRA*, *CSF1R*, *SMO*)	8 (*FGFR2*, *PDGFRA*, *ID3*, *CDH1*, *SMO*, *EPAS1*, *HIST1H3B*, *HSP90AB1*)

## Supplementary Materials

The following are available online at https://www.mdpi.com/2218-1989/10/4/144/s1, Figure S1: Illustration of simulated data, Figure S2: Error rate for the inferred network from Pearson correlation, rCCA, and rgCCA. Average of error rate for 500 simulations with 10 (**a**), 30 (**b**), 50 (**c**) and 100 (**d**) samples, Table S1: Characteristics of the TU study cohort, Table S2: Characteristics of the GU1 study cohort, Table S3: Characteristics of the GU2 study cohort, Table S4: A_XY_ covariance matrix, Table S5: B_XY_ covariance matrix, Table S6: C_XY_ covariance matrix. Section S1: Characteristics of subjects, Section S2: Simulation study to determine the correlation method for evaluating inter-omic edges, Section S3: Simulation study to determine the threshold for inter-omic edges, Section S4: Assessment of MOTA for disease classification.
