# Supplementary material for: MOTA: Network-Based Multi-Omic Data Integration for Biomarker Discovery
Source: Metabolites. 2020 Apr 8;10(4):144. doi: 10.3390/metabo10040144 (PMC7241240; doi:10.3390/metabo10040144)
Supplement: Supplementary file 1 [file metabolites-10-00144-s001.zip › metabolites-725049-supplementary.docx]

**Supplementary Material**

MOTA: Network-based multi-omic data integration for biomarker discovery

Ziling Fan, Yuan Zhou and Habtom W. Ressom *

***** Correspondence: hwr@georgetown.edu

This Supplementary Material consists of the following:

- Section S1. Characteristics of subjects
- Section S2. Simulation study to determine the correlation method for evaluating inter-omic edges.
- Section S3. Simulation study to determine the threshold for inter-omic edges
- Section S4. Assessment of MOTA for disease classification

**Section S1. Characteristics of subjects**

The samples in this study were obtained from participants recruited in Egypt and the US . Adult patients with HCC or cirrhosis recruited from the outpatient clinics and inpatient wards of the Tanta University (TU) Hospital, Egypt. In this study, these participants are referred to as TU study cohort, which consists of 89 subjects (40 HCC cases and 49 patients with liver cirrhosis). The characteristics of the patient population in the TU study cohort are summarized in **Table S1**. All participating patients provided informed consent before taking part in the study. The study protocol was approved by the Tanta University Ethical Committee. Patients were diagnosed to have cirrhosis on the basis of established clinical, laboratory and/or imaging criteria with ultrasound examination. Cases were suspected to have HCC if they had focal lesion in ultrasound examination and/or elevated AFP above 200 ng/ml, but only diagnosed to have HCC based on well-established diagnostic imaging criteria with spiral CT scanning and/or histopathology examination of liver biopsy. Clinical stages for HCC cases were determined based on the TNM staging system. Suspected cases that were not confirmed by CT or biopsy were excluded from both groups of this study. After sample collection, coded/de-identified samples were transported in dried ice to Georgetown University and stored in -80°C freezer until use. Along with the samples, we received a data set of non-identifiable information including age, gender, viral infection status, and disease stage of the study participants. We did not have access to identifying information or any information that allow to readily ascertaining the identity of the participants in the TU cohort.

The US participants in this study are adult patients recruited from the hepatology clinics at MedStar Georgetown University (GU) Hospital, Washington, DC. All patients provided informed consent and the study was approved by the Institutional Review Board at Georgetown University. Patients with liver cirrhosis were diagnosed on the basis of established clinical, laboratory and/or imaging criteria. Cases were diagnosed to have HCC based on well-established diagnostic imaging criteria and/or histology. Clinical stages for HCC cases are determined based on the TNM staging system. After collection in Georgetown University Hospital, coded/de-identified samples were stored in -80°C freezer until use. Along with the samples, we received a data set of non-identifiable information including age, gender, viral infection status, and disease stage of the study participants. In this study, these US participants are split into two cohorts and are referred to as GU1 and GU2 study cohorts. The participants in the GU1 study cohort consist of 94 subjects (48 HCC cases and 46 patients with liver cirrhosis). The characteristics of the GU1 study cohort are summarized in **Table S2**. The participants in the GU2 cohort consist of 65 subjects (40 HCC cases and 25 patients with liver cirrhosis). **Table S3** provides the characteristics of the GU2 study cohort.

**Table S1. Characteristics of the TU study cohort.**

|  |  | **HCC (*n*=40)** | **Cirrhosis (*n*=49)** | ***p*-value** |
| --- | --- | --- | --- | --- |
| **Age** | Mean (SD) | 53.2 (3.9) | 53.8 (7.6) | 0.3530 |
|  |  |  |  |  |
| **BMI** | Mean (SD) | 24.9 (3.1) | 24.5 (4.4) | 0.6513 |
|  |  |  |  |  |
| **Gender** | Male | 77.5% | 67.3% | 0.3474 |
|  |  |  |  |  |
| **HCV serology** | HCV Ab+ | 100.0% | 100.0% | 1.0000 |
|  |  |  |  |  |
| **HBV serology** | HBsAg+ | 0.0% | 6.1% | 0.2492 |
|  |  |  |  |  |
| **MELD** | Mean (SD) | 18.6 (7.7) | 18.9 (7.1) | 0.1328 |
|  | MELD $\leq$ 10 | 20.0% | 12.2% | 0.3863 |
|  |  |  |  |  |
| **Child-Pugh grade** | A | 15.0% | 0% | 0.0117 |
|  | B | 47.5% | 46.9% |  |
|  | C | 37.5% | 53.1% |  |
|  |  |  |  |  |
| **AFP** | Median (IQR) | 275.9 (1244.3) |  |  |
|  |  |  |  |  |
| **HCC stage** | Stage I | 72.5% |  |  |
|  | Stage II | 15.0% |  |  |
|  | Stage III | 5.0% |  |  |
|  | Unknown | 7.5% |  |  |

**Table S2. Characteristics of the GU1 study cohort.**

|  |  | **HCC (*n*=48)** | **Cirrhosis (*n*=46)** | ***p*-value** |
| --- | --- | --- | --- | --- |
| **Age** | Mean (SD) | 60.2 (6.0) | 58.9 (7.1) | 0.3443 |
|  |  |  |  |  |
| **BMI** | Mean (SD) | 29.6 (6.1) | 29.4 (6.9) | 0.8978 |
|  |  |  |  |  |
| **Gender** | Male | 77.1% | 73.9% | 0.8121 |
|  |  |  |  |  |
| **Ethnicity** | Caucasian | 50.0% | 63.0% | 0.4566 |
|  | African American | 33.3% | 26.1% |  |
|  | Others | 16.7% | 10.9% |  |
|  |  |  |  |  |
| **HCV serology** | HCV Ab+ | 68.8% | 41.3% | 0.0033 |
|  | HCV RNA+ | 62.5% | 39.1% | 0.0385 |
|  |  |  |  |  |
| **HBV serology** | Anti HBC+ | 45.8% | 26.1% | 0.0554 |
|  | HBsAg+ | 8.3% | 2.2% | 0.3619 |
|  |  |  |  |  |
| **MELD** | Mean (SD) | 11.3 (4.1) | 17.3 (16.1) | 0.0190 |
|  | MELD ≤ 10 | 47.9% | 10.9% | 0.0042 |
|  |  |  |  |  |
| **Child-Pugh grade*** | A | 46.3% | 13.0% | 0.0008 |
|  | B | 43.9% | 54.3% |  |
|  | C | 9.8% | 32.6% |  |
|  |  |  |  |  |
| **AFP** | Median (IQR) | 38.8 (91.1) | 4.5 (11.85) | 0.0001 |
|  |  |  |  |  |
| **HCC stage** | Stage I | 54.2% |  |  |
|  | Stage II | 22.9% |  |  |
|  | Stage III | 6.3% |  |  |
|  | Unknown | 16.7% |  |  |

*Child-Pugh grade is unknown in seven HCC patients.

**Table S3. Characteristics of the GU2 study cohort.**


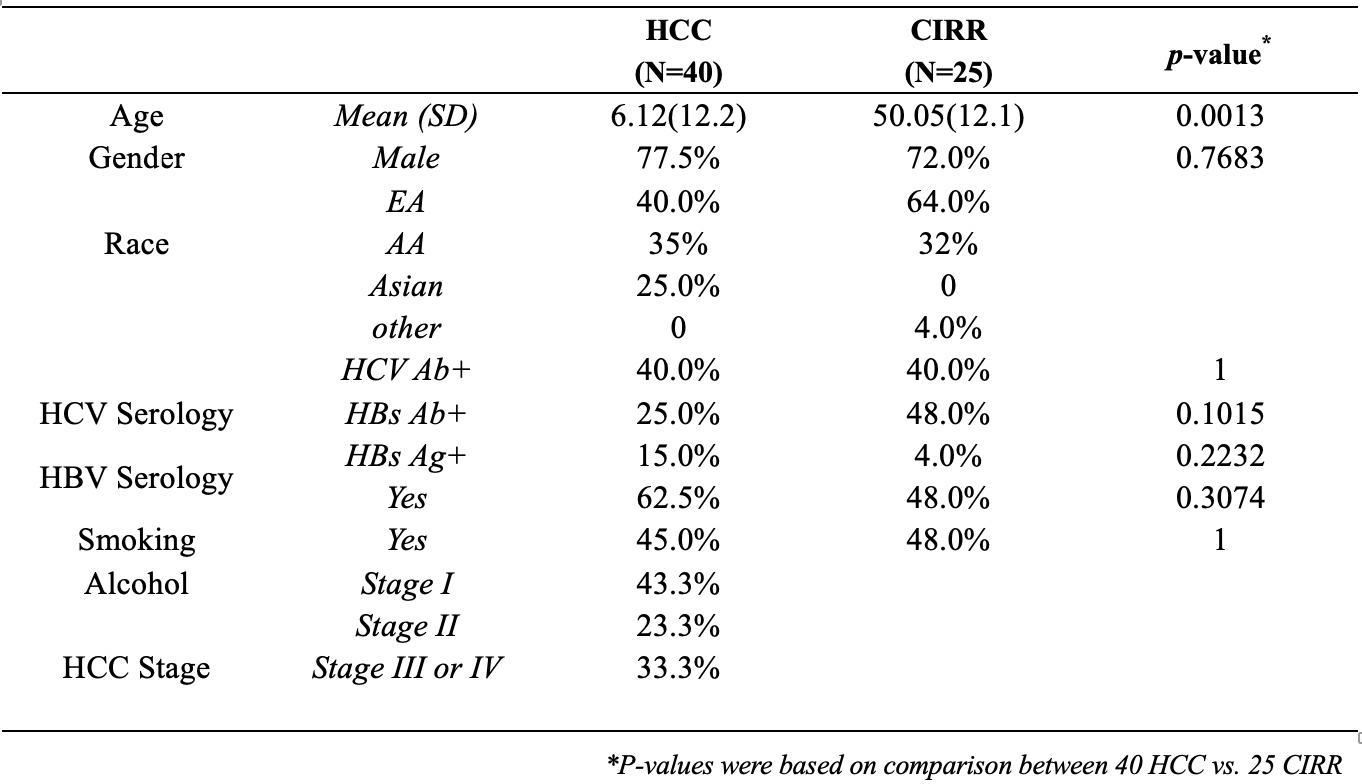


**Section S2. Simulation study to determine the correlation method for evaluating inter-omic edges.**

In order to determine the correlation method that is best suited for evaluating inter-omic connections, we performed a simulation study. Specifically, we compared the performance of Pearson correlation, rCCA, and rgCCA for inferring correlations of features in different datasets. This was accomplished by generated three simulation datasets ($\boldsymbol{X}$, $\boldsymbol{Y}$ and $\boldsymbol{Z})$ with 100 features/variables and sample sizes of 10, 30, 50 and 100 for all three data sets. We assumed that only $\boldsymbol{X}$, $\boldsymbol{Y}$ and $\boldsymbol{X}$, $\boldsymbol{Z}$ have associations. A subset of relevant variables in $X$ is associated with a subset of relevant variables in $\boldsymbol{Y}$ and $\boldsymbol{Z}$ according to the model described below, and the remaining variables are simulated as noises.

1. Subsets of relevant variables from $\boldsymbol{X}$ and $\boldsymbol{Y}$ datasets were generated with covariance matrix ∑ defined by:

$=\left[ \begin{matrix} {}_{XX} & {}_{XY} \\ {}_{XY}^{'} & {}_{YY} \end{matrix} \right]$, with ${}_{XY}$ = $\left[ \begin{matrix} \boldsymbol{A}_{XY} & 0 & 0 \\ 0 & \boldsymbol{B}_{XY} & 0 \\ 0 & 0 & \boldsymbol{C}_{XY} \end{matrix} \right]$

Relevant variables from Z dataset were generated using the same method as described above.

1. $\boldsymbol{X}$ consists of three independent sets with 10, 10 and 3 variables and they are annotated as $\boldsymbol{X}_{A}=\left[ \boldsymbol{x}_{A}^{1},\cdots,\boldsymbol{x}_{A}^{10} \right]$, $\boldsymbol{X}_{B}=\left[ \boldsymbol{x}_{B}^{1},\cdots,\boldsymbol{x}_{B}^{10} \right]$ and $\boldsymbol{X}_{C}=\left[ \boldsymbol{x}_{C}^{1},\boldsymbol{x}_{C}^{2},\boldsymbol{x}_{C}^{3} \right]$.
2. $\boldsymbol{Y}$ consists of three independent sets with 10, 5 and 2 variables and they are annotated as $\boldsymbol{Y}_{A}=\left[ \boldsymbol{y}_{A}^{1},\cdots,\boldsymbol{y}_{A}^{10} \right]$, $\boldsymbol{Y}_{B}=\left[ \boldsymbol{y}_{B}^{1},\cdots,\boldsymbol{y}_{B}^{5} \right]$ and $\boldsymbol{Y}_{C}=\left[ \boldsymbol{y}_{C}^{1},\boldsymbol{y}_{C}^{2} \right]$. $\boldsymbol{Z}$ has the same structure as $\boldsymbol{Y}$ and annotated as $\boldsymbol{Z}_{A}=\left[ \boldsymbol{z}_{A}^{1},\cdots,\boldsymbol{z}_{A}^{10} \right]$, $\boldsymbol{Z}_{B}=\left[ \boldsymbol{z}_{B}^{1},\cdots,\boldsymbol{z}_{B}^{5} \right]$ and $\boldsymbol{Z}_{C}=\left[ \boldsymbol{z}_{C}^{1},\boldsymbol{z}_{C}^{2} \right]$.
3. The relevant variables in $\boldsymbol{X}_{A}$ and $\boldsymbol{Y}_{A}$ were generated with a negative cross-correlation with correlation coefficient in the range of -0.91 to -0.61. The variables in $\boldsymbol{X}_{B}$ and $\boldsymbol{Y}_{B}$ were generated with a positive cross-correlation with correlation coefficient in the range of 0.53 to 0.95. The variables in $\boldsymbol{X}_{C}$ and $\boldsymbol{Y}_{C}$ were generated with an absolute cross-correlation coefficient in the range of 0.81 to 0.93. $\boldsymbol{Z}_{A}{, \boldsymbol{Z}}_{B}$ and $\boldsymbol{Z}_{C}$ share the same correlational relationship with the variables in ***X*** with variables in $\boldsymbol{Y}$ .
4. The noise variables were generated assuming normal distributions with zero mean and variance one. The noise variables were appended to the subsets which described above such that final data set consist of 100 variables for all $\boldsymbol{X}$, $\boldsymbol{Y}$ and $\boldsymbol{Z}$.

**Cross-covariance matrix between** $\boldsymbol{X}$ **and** $\mathbf{Y}$

${}_{XY}$ = $\left[ \begin{matrix} \boldsymbol{A}_{XY} & 0 & 0 \\ 0 & \boldsymbol{B}_{XY} & 0 \\ 0 & 0 & \boldsymbol{C}_{XY} \end{matrix} \right]$

where one simulation example of $\boldsymbol{A}_{XY}$, $\boldsymbol{B}_{XY}$ and $\boldsymbol{C}_{XY}$ are given in Tables S4, S5, and S6, respectively. Covariance matrix between ***X*** and ***Z*** share a similar pattern with different number of variables.

Table S4. $\boldsymbol{A}_{XY}$covariance matrix

|  | $\boldsymbol{x}_{A}^{1}$ | $\boldsymbol{x}_{A}^{2}$ | $\boldsymbol{x}_{A}^{3}$ | $\boldsymbol{x}_{A}^{4}$ | $\boldsymbol{x}_{A}^{5}$ | $\boldsymbol{x}_{A}^{6}$ | $\boldsymbol{x}_{A}^{7}$ | $\boldsymbol{x}_{A}^{8}$ | $\boldsymbol{x}_{A}^{9}$ | $\boldsymbol{x}_{A}^{10}$ |
| --- | --- | --- | --- | --- | --- | --- | --- | --- | --- | --- |
| $\boldsymbol{y}_{A}^{1}$ | -0.92 | -0.76 | -0.79 | -0.84 | -0.76 | -0.80 | -0.78 | -0.85 | -0.80 | -0.69 |
| $\boldsymbol{y}_{A}^{2}$ | -0.88 | -0.86 | -0.76 | -0.80 | -0.72 | -0.79 | -0.81 | -0.76 | -0.70 | -0.68 |
| $\boldsymbol{y}_{A}^{3}$ | -0.74 | -0.72 | -0.89 | -0.83 | -0.69 | -0.79 | -0.80 | -0.81 | -0.75 | -0.72 |
| $\boldsymbol{y}_{A}^{4}$ | -0.72 | -0.69 | -0.81 | -0.92 | -0.66 | -0.77 | -0.72 | -0.82 | -0.70 | -0.80 |
| $\boldsymbol{y}_{A}^{5}$ | -0.78 | -0.66 | -0.79 | -0.81 | -0.87 | -0.74 | -0.71 | -0.87 | -0.82 | -0.76 |
| $\boldsymbol{y}_{A}^{6}$ | -0.82 | -0.76 | -0.76 | -0.81 | -0.68 | -0.90 | -0.72 | -0.78 | -0.74 | -0.76 |
| $\boldsymbol{y}_{A}^{7}$ | -0.82 | -0.74 | -0.84 | -0.79 | -0.75 | -0.71 | -0.86 | -0.80 | -0.73 | -0.66 |
| $\boldsymbol{y}_{A}^{8}$ | -0.79 | -0.68 | -0.73 | -0.79 | -0.72 | -0.77 | -0.77 | -0.84 | -0.84 | -0.71 |
| $\boldsymbol{y}_{A}^{9}$ | -0.84 | -0.72 | -0.78 | -0.82 | -0.82 | -0.83 | -0.74 | -0.84 | -0.90 | -0.70 |
| $\boldsymbol{y}_{A}^{10}$ | -0.76 | -0.73 | -0.78 | -0.74 | -0.73 | -0.81 | -0.77 | -0.77 | -0.78 | -0.84 |

Table S5. $\boldsymbol{B}_{XY}$ covariance matrix

|  | $\boldsymbol{x}_{B}^{1}$ | $\boldsymbol{x}_{B}^{2}$ | $\boldsymbol{x}_{B}^{3}$ | $\boldsymbol{x}_{B}^{4}$ | $\boldsymbol{x}_{B}^{5}$ |
| --- | --- | --- | --- | --- | --- |
| $\boldsymbol{x}_{B}^{1}$ | 0.91 | 0.68 | 0.82 | 0.79 | 0.77 |
| $\boldsymbol{x}_{B}^{2}$ | 0.80 | 0.87 | 0.77 | 0.78 | 0.83 |
| $\boldsymbol{x}_{B}^{3}$ | 0.78 | 0.66 | 0.91 | 0.75 | 0.78 |
| $\boldsymbol{x}_{B}^{4}$ | 0.78 | 0.63 | 0.77 | 0.87 | 0.69 |
| $\boldsymbol{x}_{B}^{5}$ | 0.73 | 0.78 | 0.82 | 0.75 | 0.94 |
| $\boldsymbol{x}_{B}^{6}$ | 0.76 | 0.63 | 0.82 | 0.79 | 0.73 |
| $\boldsymbol{x}_{B}^{7}$ | 0.79 | 0.67 | 0.81 | 0.78 | 0.73 |
| $\boldsymbol{x}_{B}^{8}$ | 0.82 | 0.67 | 0.81 | 0.71 | 0.69 |
| $\boldsymbol{x}_{B}^{9}$ | 0.83 | 0.69 | 0.77 | 0.69 | 0.64 |
| $\boldsymbol{x}_{B}^{10}$ | 0.80 | 0.63 | 0.90 | 0.72 | 0.70 |

Table S6. $\boldsymbol{C}_{XY}$ covariance matrix

|  | $\boldsymbol{x}_{C}^{1}$ | $\boldsymbol{x}_{C}^{2}$ |
| --- | --- | --- |
| $\boldsymbol{y}_{C}^{1}$ | 0.92 | -0.82 |
| $\boldsymbol{y}_{C}^{2}$ | 0.82 | -0.87 |
| $\boldsymbol{y}_{C}^{3}$ | 0.82 | -0.83 |


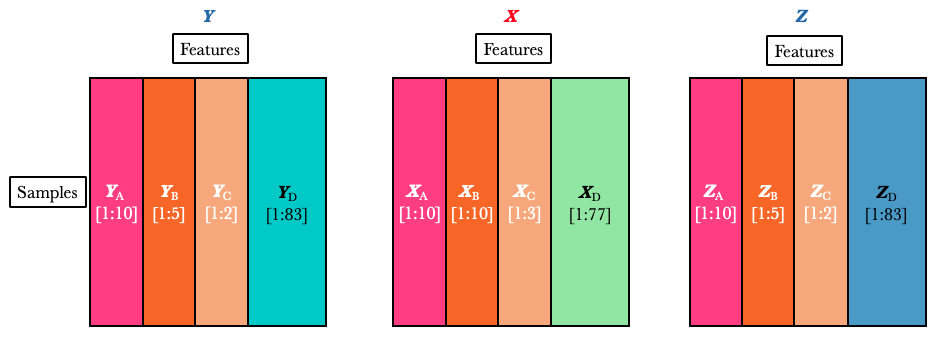


**Figure S1.** Illustration of simulated data. Only associations of features in $\boldsymbol{X}$&$\boldsymbol{Y}$ and $\boldsymbol{X}$&$\boldsymbol{Z}$ are considered. The features in blocks with the same color (A, B and C) are associated and D blocks are noise variables with no association. Any two features across the same color blocks have an association and corresponding correlations. Note that we only consider associations across different datasets rather than within the same dataset. Specially, the relevant variables in $\boldsymbol{X}_{A}$ and $\boldsymbol{Y}_{A}$ have negative correlation (-0.91 ~ -0.61), the relevant variables in $\boldsymbol{X}_{B}$ and $\mathbf{Y}_{B}$ have been assigned positive correlation (0.53~0.95) and relevant variables in $\mathbf{X}_{\mathbf{c}}$ and $\mathbf{Y}_{c}$ have positive or negative correlation (absolute correlation 0.81 ~ 0.93). **Z** dataset was generated using the same idea as $\boldsymbol{Y}$ . $\boldsymbol{X}_{D}$,$\boldsymbol{Y}_{D}$,$\boldsymbol{Z}_{D}$ are noise variables generated assuming normal distributions with zero mean and variance one.

We adopted the same method as in ref. [21] to draw the error curve to compare three methods (Pearson, rCCA and rgCCA) to infer inter-omic connections. Specially, the correlation matrices are calculated using the three methods and a series of ‘signed’ adjacency matrices (1 or -1 indicates that the absolute value of correlation between two variables are higher than the threshold and 0 indicates that the absolute value of correlation between two variables are lower than the threshold) are generated according to a threshold ranging from 0 to 1 with a step of 0.01 and each inferred adjacency matrix is annotated as ***A***. Based on the ground truth adjacency matrix ***G***, the error rate is calculated using the equation below.

$$\sum\frac{\left| \mathbf{G}-\boldsymbol{A} \right|}{n}$$

where $n$ is the number of elements in correlation matrix. Each error rate is calculated based on the average from 500 simulations.


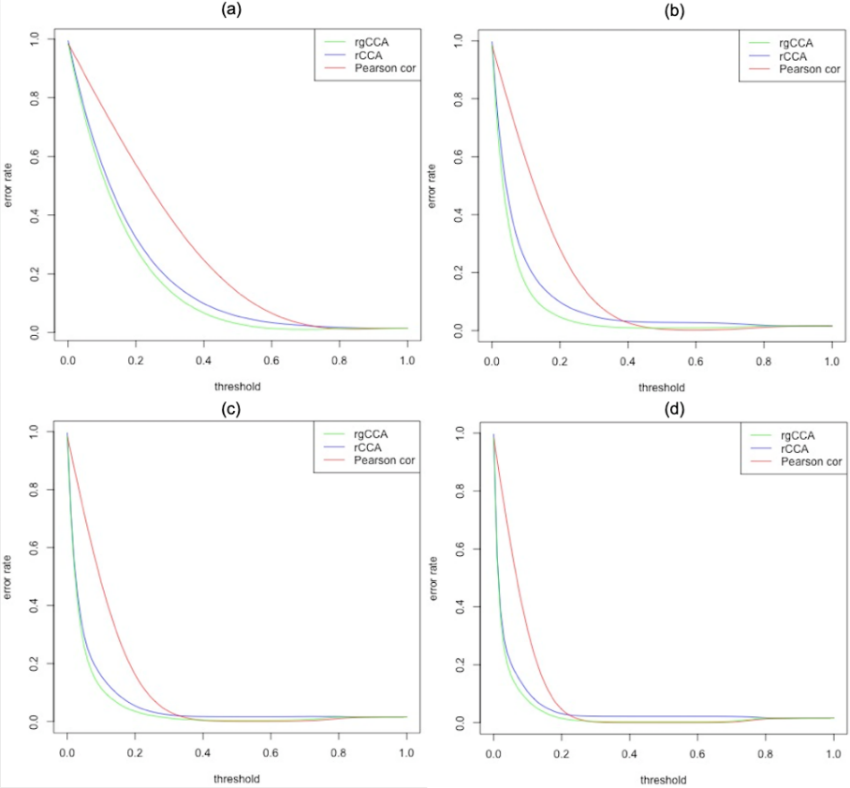


**Figure S2.** Error rate for the inferred network from Pearson correlation, rCCA, and rgCCA. Average of error rate for 500 simulations with 10 (a), 30 (b), 50 (c) and 100(d) samples.

Figure S2 shows that rgCCA achieves the lowest error rate in all of four simulation setups regardless of the threshold level. Therefore, not only rgCCA overcomes the limitation of rCCA but also it has a better performance for inferring inter-omic connections.

From Figure S2, it can be observed that small sample size tends to increase the error rate of network inference. We expect further increase of error rate when the number of variables gets larger.

**Section S3. Simulation study to determine the threshold for inter-omic edges**

To determine inter-omic connections using differential canonical correlation, we had to specify a cutoff that determines where or not an edge connecting two nodes should be added. A 0.5 cutoff led to the creation of 39 inter-omic edges for the GU1 dataset and 109 edges for the TU dataset. These represent 0.4% of all possible edges (9,699 possible edges) and 0.9% (12,012 possible edges) for the GU1 and TU datasets, respectively. Analysis of the data by MOTA led to four overlapping metabolites between the GU1, TU and GU1&TU combined datasets with relatively high ranking for tyrosine and α-TOH. A 0.4 cutoff increased the inter-omic connections to 313 for GU1 dataset and to 549 for the TU dataset, which constitute about 3.2% and 4.6% of all possible edges, respectively. However, four overlapping metabolites were ranked by MOTA in the top 10 by MOTA in both datasets with tyramine replaced by glycine. Tyramine disappeared from the top 10 ranked list in the GU1 dataset and glycine’s ranking increased to 9^th^ in the TU dataset. The reason for glycine’s ranking increase is that it connects more glycans after lowering the cutoff. As shown in Figure 3, there are edges connecting glycine with three glycans in the GU1 network, although these glycans do not have low p-values. For potential MOTA users, we recommend that they determine the cutoff starting from calculating the differential correlation for all possible edges. Then, 0.5% of all possible edges could be created if a strict cutoff is desired and 5% of all possible edges could be created if a relatively loose cutoff is desired. With the information, a data-set specific cutoff could be determined.

**Section S4.** Assessment of MOTA for disease classification

We evaluated the disease classification ability of features selected by MOTA. Specifically, we trained a Random Forest model using the top 10 metabolite features ranked by student t-test and MOTA from the TU dataset and did 5 fold-cross validation on the same dataset. The results show that AUC for model trained by features ranked by MOTA and student t-test is 0.64 and 0.69 respectively. Next, we trained another Random Forest model using the features selected above (top 10 metabolite features by the two methods from TU cohort) using GU1 dataset and did five-fold cross-validation. The results show that the model trained using features ranked by MOTA outperforms those selected by student t-test by a small margin, with AUC of 0.66 and 0.64 respectively. Comparing two sets of results, features ranked by MOTA from the TU dataset maintain their classification ability when applying them on the GU1 dataset (0.64 vs 0.66). However, features ranked by student t-test dropped by 0.05 from 0.69 to 0.64. These results indicate that features ranked by combining differential network topology and statistical significance of changes in biomolecule levels lead to more consistent performance in different cohorts of the same study than those selected by merely network topology or statistical significance.
